# Remarkable Plasticity of Bone Iron Homeostasis in Hibernating Daurian Ground Squirrels (*Spermophilus dauricus*) May Be Involved in Bone Maintenance

**DOI:** 10.3390/ijms232415858

**Published:** 2022-12-13

**Authors:** Yue He, Yong Kong, Rongrong Yin, Huajian Yang, Jie Zhang, Huiping Wang, Yunfang Gao

**Affiliations:** 1 Shaanxi Key Laboratory for Animal Conservation, College of Life Sciences, Northwest University, Xi’an 710069, China; 2 Key Laboratory of Resource Biology and Biotechnology in Western China, Ministry of Education, Northwest University, Xi’an 710069, China

**Keywords:** Daurian ground squirrels, hibernation, femur, iron metabolism

## Abstract

Iron overload is an independent risk factor for disuse osteoporosis. Hibernating animals are natural models of anti-disuse osteoporosis; however, whether iron metabolism is involved in bone adaptation and maintenance during hibernation is unclear. To investigate this question, Daurian ground squirrels (*Spermophilus dauricus*) (*n* = 5–6/group) were used to study changes in bone iron metabolism and its possible role in anti-disuse osteoporosis during hibernation. Iron content in the femur and liver first decreased in the torpor group (vs. summer group, −66.8% and −25.8%, respectively), then recovered in the post-hibernation group, suggesting remarkable plasticity of bone iron content. The expression of ferritin in the femur and hepcidin in the liver also initially decreased in the torpor group (vs. summer group, −28.5% and −38.8%, respectively), then increased in the inter-bout arousal (vs. torpor group, 126.2% and 58.4%, respectively) and post-hibernation groups (vs. torpor group, 153.1% and 27.1%, respectively). In conclusion, bone iron metabolism in hibernating Daurian ground squirrels showed remarkable plasticity, which may be a potential mechanism to avoid disuse bone loss during extended periods of inactivity. However, the specific location of iron during low-iron hibernation and the source of iron in post-hibernation recovery need to be further explored.

## 1. Introduction

**Figure 1:**
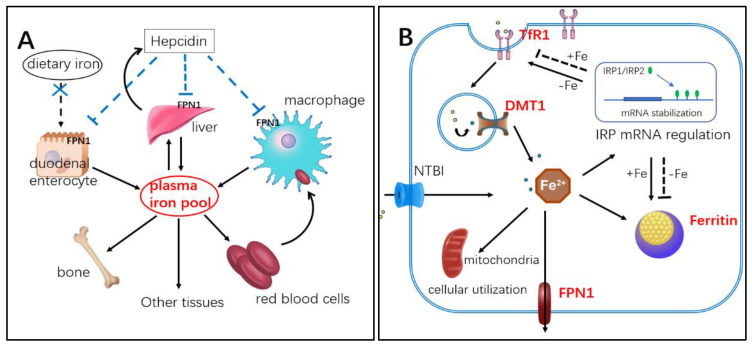
Schematic of iron metabolism. (**A**) Systemic iron metabolism: hepcidin is secreted by the liver, which inhibits expression of iron-releasing protein ferroportin 1 (FPN1) on membrane of intestinal epithelial cells, liver, and macrophages, thus controlling serum iron concentration. (**B**) Cellular iron metabolism: Transferrin receptor 1 (TfR1) on the surface of the cell membrane binds to iron-containing transferrin in blood and is ingested into the cell via endocytosis. Ferric iron then breaks away from the receptor and is converted to ferrous iron, then released into the cytoplasm through divalent metal transporter 1 (DMT1). Some iron in the cytoplasm is utilized biologically, some is stored after binding with ferritin, and some is excreted by FPN1. When intracellular iron content is low (-Fe), ferritin synthesis is inhibited by iron regulatory protein (IRP) regulation, while TfR1 expression is increased, and iron absorption capacity is enhanced.

Iron is an indispensable trace element, which plays an important role in many physiological and biochemical processes in the body, such as oxygen transport, DNA synthesis, electron transport, and enzyme catalysis. However, when iron exceeds the physiological requirements of the body, abnormal deposits can occur in tissues and organs, resulting in iron overload toxicity. Therefore, it is necessary to control iron content at the cellular and systemic levels (See schematic of iron metabolism in Figure 1A,B). Iron overload toxicity occurs primarily through the generation of hydroxyl radicals by excess free iron and hydrogen peroxide interactions (Fenton reaction). These reactive oxygen species (ROS) can oxidize many biomolecules in the body, including lipids, carbohydrates, proteins, and DNA, and disrupt normal biological functions [1,2]. Iron overload also has potential effects on various diseases, including cancer [3], cardiovascular disease [4], diabetes [5], and neurodegeneration [6].

Recent studies have shown that iron overload is also a risk factor for osteoporosis, which is closely associated with bone loss. Iron overload can activate oxidative stress in the body, up-regulate osteoclast differentiation, enhance bone resorption, inhibit osteoblast expression, and reduce bone formation [7,8,9]. Iron overload may also directly lead to osteoporosis by down-regulating the expression of osteoblast-specific genes and inhibiting the growth of hydroxyapatite crystals [10,11]. Animal models provide further evidence of the harmful effects of excess iron on bone health. For example, iron overload in mice treated with high doses of iron dextran results in ROS production, bone resorption, bone structure damage, and bone loss [12]. In contrast, iron chelator deferoxamine mesylate (DFO) treatment in mice reduces iron content in bone and reduces bone loss caused by de-loading [13]. Clinical studies and case reports have also found that patients with iron overload due to hemodialysis, menopause, and aging are more likely to develop bone abnormalities, suggesting that iron overload is an independent risk factor for osteoporosis [14]. Thus, these in vitro and in vivo studies provide evidence for the harmful effects of iron overload on bone metabolism.

Physical unloading and prolonged bed rest can lead to disuse osteoporosis [15], and disuse-induced bone loss is strongly associated with changes in iron metabolism [16]. Yang et al. reported significant iron accumulation in the femur, liver, spleen, and serum as well as severe loss of femoral microstructure and bone mineral density after 4 weeks of hindlimb unloading in mice [17]. Astronauts experience abnormal iron-to-tissue migration during spaceflight, with increased bone iron reserves and decreased bone mineral density, suggesting that increased bone iron reserves may be a risk factor for bone resorption [18]. In addition, humans show an increase in iron storage, bone iron levels, oxidative damage markers, and bone resorption after 2 months in bed [19,20]. These reports suggest that iron overload may be involved in the development of disuse osteoporosis under conditions of weightlessness, mechanical unloading, and lack of physical activity.

Hibernating animals undergo long periods of inactivity (4–8 months) each year. However, several studies indicate that hibernating animals, e.g., black bears (*Ursus americanus*) [21], yellow-bellied marmots (*Marmota flaviventris*) [22,23], and thirteen-lined ground squirrels (*Ictidomys tridecemlineatus*) [24], show good resistance to disuse bone loss after prolonged hibernation inactivity. Considering that iron overload is a high-risk factor for osteoporosis, we hypothesize that hibernating animals may resist disuse osteoporosis due to inactivity by avoiding iron overload.

To investigate changes in bone iron metabolism during hibernation and its possible role in anti-disuse osteoporosis, we studied changes in femur microstructure and mechanical strength, femur and liver iron content, and iron-metabolism-related protein expression in Daurian ground squirrels (*Spermophilus dauricus*) during the summer and hibernation periods (pre-hibernation, torpor, interbout arousal, near post-hibernation, and post-hibernation) (Figure S1). To the best of our knowledge, this is the first study to explore the potential mechanism of iron metabolism in resisting disuse (extended periods of inactivity) bone loss in Daurian ground squirrels throughout the entire hibernation process.

## 2. Results

### 2.1. Bone Mass, Mechanical Properties, and Microstructure

**Figure 2:**
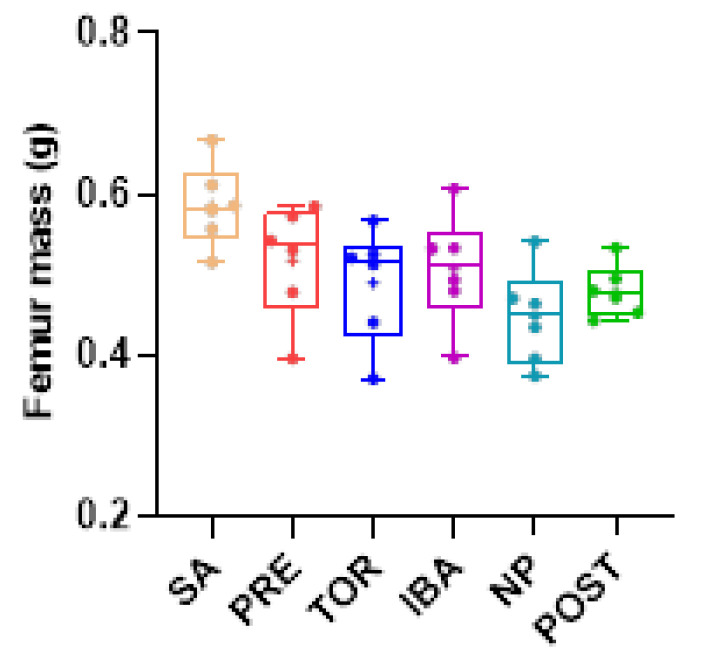
Comparison of femur mass in Daurian ground squirrels in different groups. Boxes represent upper and lower quartiles, middle horizontal line represents median, plus sign represents average, lines extending from upper and lower ends represent upper and lower edges, respectively, and points represent individual sample values (*n* = 6). No superscript letters denote no significant differences among groups by Sidak test (*p* > 0.05). SA, summer active group; PRE, pre-hibernation group; TOR, torpor group; IBA, interbout arousal group; NP, near post-hibernation group; and POST, post-hibernation group.

**Figure 3:**
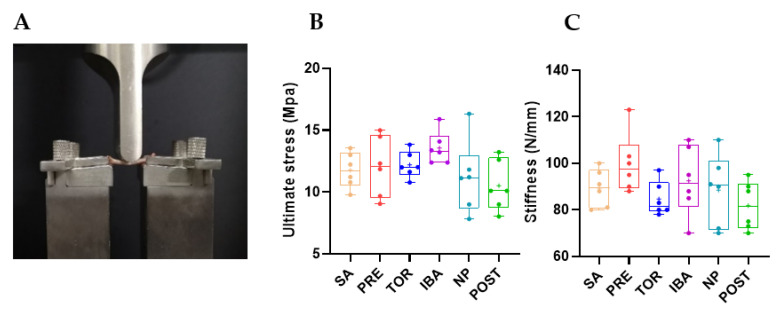
Comparison of mechanical properties of femur in different groups. (**A**) Experimental set-up for biomechanical testing, (**B**) Ultimate stress, and (**C**) Stiffness. Boxes represent upper and lower quartiles, middle horizontal line represents median, plus sign represents average, lines extending from upper and lower ends represent upper and lower edges, respectively, and points represent individual sample values (*n* = 6). No superscript letters denote no significant differences among groups by Sidak test (*p* > 0.05). SA, summer active group; PRE, pre-hibernation group; TOR, torpor group; IBA, interbout arousal group; NP, near post-hibernation group; and POST, post-hibernation group.

**Figure 4:**
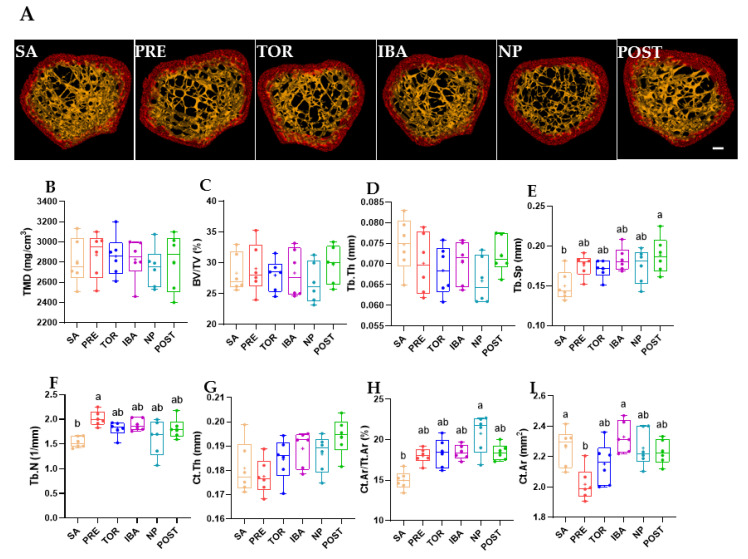
Changes in microstructural parameters of femur in different groups. (**A**) Representative micro-CT images of distal femur in different groups (Bar = 200 μm). Related parameters: X-ray tube potential of 50 kVp and tube current of 80 μA in air. The captured images were acquired at a voxel size of 43 μm (filter: Cu 0.06 mm + Al 0.5 mm). Region of interest (ROI) was defined as the coronal central region of the distal femur (0.5 mm thick; number of slices: 512 layers). Histograms of microstructural parameters, including (**B**) tissue mineral density (TMD, mg/cm^3^), (**C**) bone volume fraction (BV/TV, %), (**D**) trabecular thickness (Tb.Th, mm), (**E**) trabecular separation (Tb.Sp, mm), (**F**) trabecular number (Tb.N, 1/mm), (**G**) average cortical thickness (Ct.Th, mm), (**H**) cortical area fraction (Ct.Ar/Tt.Ar, %), and (**I**) cortical area (Ct.Ar, mm^2^) in distal femur of different groups. SA, summer active group; PRE, pre-hibernation group; TOR, torpor group; IBA, interbout arousal group; NP, near post-hibernation group; and POST, post-hibernation group. Boxes represent upper and lower quartiles, middle horizontal line represents median, plus sign represents average, lines extending from upper and lower ends represent upper and lower edges, respectively, and points represent individual sample values (*n* = 6). Superscripts with different letters indicate significant differences (*p* < 0.05) among groups by Sidak test. Superscripts with same letter or no superscripts denote no significant differences (*p* > 0.05).

Femur mass of Daurian ground squirrels did not change significantly in the different hibernation periods (no superscript letters, *p* > 0.05, Figure 2), nor did ultimate stress (the maximum stress on bone before deformation) or stiffness (the slope of the load–deformation curve) (*p* > 0.05, Figure 3B,C), suggesting that bone mass and mechanical properties were effectively maintained during long-term inactivity. The micro-CT results (Table S1) showed that compared with the SA group, Tb.Sp, Tb.N, and Ct.Ar/Tt.Ar were significantly higher in the POST, PRE, and NP groups, respectively (different superscript letters, *p* = 0.006, *p* = 0.005, and *p* < 0.001, respectively, Figure 4E,F,H). Ct.Ar was higher in the SA and IBA groups compared with the PRE group (*p* = 0.021, *p* = 0.027, respectively, Figure 4I), but there were no marked differences in other indices among the groups (*p* > 0.05, Figure 4B–D,G). Thus, while trabecular bone loss occurred in the squirrels during hibernation inactivity, the other cortical and cancellous bone parameters did not change significantly.

### 2.2. Total Iron Content in Femur and Liver during Different Periods

**Figure 5:**
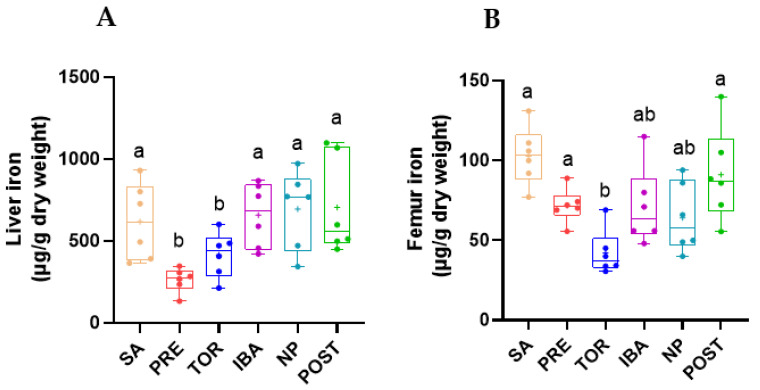
Changes in iron content in liver (**A**) and femur (**B**) of Daurian ground squirrels in different groups. SA, summer active group; PRE, pre-hibernation group; TOR, torpor group; IBA, interbout arousal group; NP, near post-hibernation group; and POST, post-hibernation group. Boxes represent upper and lower quartiles, middle horizontal line represents median, plus sign represents average, lines extending from upper and lower ends represent upper and lower edges, respectively, and points represent individual sample values (*n* = 6). Superscripts with different letters indicate significant differences (*p* < 0.05) among groups by Sidak test. Superscripts with same letters denote no significant differences (*p* > 0.05).

Iron content in the liver and femur of Daurian ground squirrels was detected by iCAP. Compared with the SA group, iron content in the liver was low in the PRE group (−51.8%) and remained low in the TOR group (−31.4%). Iron content in the PRE and TOR groups was significantly lower than that in the other groups (PRE vs. SA, IBA, NP, POST; *p* = 0.001, *p* = 0.001, *p* < 0.001, and *p* < 0.001, respectively; TOR vs. SA, IBA, NP, POST; *p* = 0.017, *p* = 0.004, *p* = 0.008, and *p* = 0.012, respectively) (Figure 5A). Iron content in the femur was significantly lower in the TOR group than in the SA, PRE, and POST groups (−66.8%, −41.9%, and −45.8%, respectively; *p* < 0.001, *p* = 0.026, and *p* = 0.001, respectively) (Figure 5B). These results suggest that Daurian ground squirrels may regulate iron content in tissues and maintain relatively low iron levels during hibernation.

### 2.3. Iron Distribution in Femur and Liver during Different Periods

**Figure 6:**
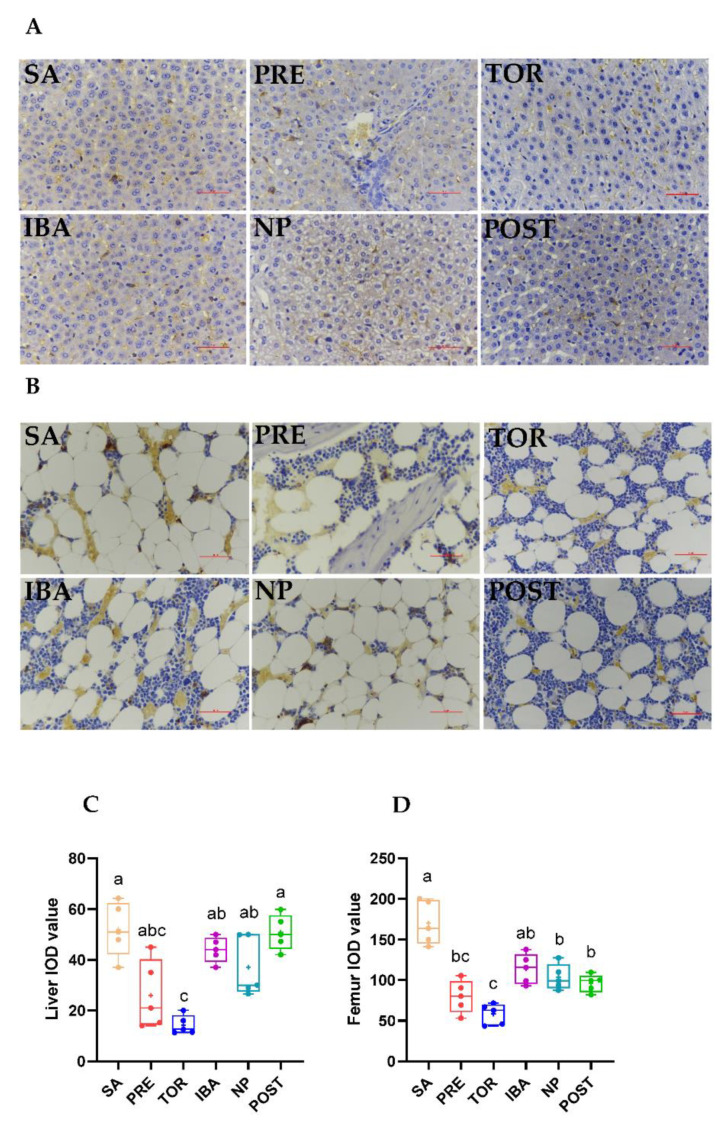
Differences in iron distribution in liver and femur of Daurian ground squirrels in different groups. Histological analysis of iron deposits in liver (**A**, Bar = 50 μm) and femur (**B**, Bar = 50 μm, represents the actual length of the image) sections by DAB-Prussian iron staining. (**C**,**D**) Integrated optical density (IOD) of iron-positive particles in liver and femur, respectively. SA, summer active group; PRE, pre-hibernation group; TOR, torpor group; IBA, interbout arousal group; NP, near post-hibernation group; and POST, post-hibernation group. Boxes represent upper and lower quartiles, middle horizontal line represents median, plus sign represents average, lines extending from upper and lower ends represent upper and lower edges, respectively, and points represent individual sample values (*n* = 5). Superscripts with different letters indicate significant differences (*p* < 0.05) among groups by Sidak test. Superscripts with same letters denote no significant differences (*p* > 0.05).

Prussian blue staining was performed on liver and femur sections to detect iron deposition. Results showed that iron distribution in the liver and femur was consistent with iron content in the corresponding tissues. The positive staining area of the liver in the TOR group was significantly lower than that in the SA and POST groups (vs. SA, −75.4%; vs. POST, −74.2%, Figure 6C, *p* = 0.01, *p* < 0.001, respectively). The positive area in the femur was significantly lower in the PRE, TOR, NP, and POST groups than in the SA group (−57.8%, −64.4%, −37.3%, and −41.9%, respectively; *p* < 0.001, *p* = 0.001, *p* < 0.001, and *p* < 0.001, respectively) and the positive area was significantly higher in the IBA, NP, and POST groups than in the TOR group (*p* = 0.001, *p* = 0.008, and *p* = 0.042, respectively; Figure 6D).

### 2.4. Regulation of Hepcidin in Liver during Different Periods

**Figure 7:**
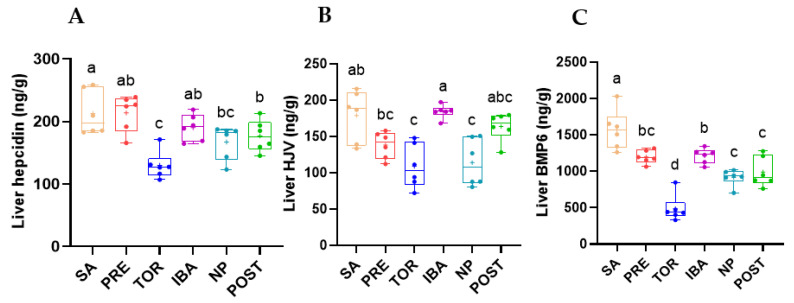
Changes in expression of hepcidin (**A**), hemojuvelin (HJV) (**B**), and bone morphogenetic protein 6 (BMP-6) (**C**) in liver in different groups. SA, summer active group; PRE, pre-hibernation group; TOR, torpor group; IBA, interbout arousal group; NP, near post-hibernation group; and POST, post-hibernation group. Boxes represent upper and lower quartiles, middle horizontal line represents median, plus sign represents average, lines extending from upper and lower ends represent upper and lower edges, respectively, and points represent individual sample values (*n* = 6). Superscripts with different letters indicate significant differences (*p* < 0.05) among groups by Tamhane or Sidak test. Superscripts with same letters denote no significant differences (*p* > 0.05).

The expression level of hepcidin in the liver of the TOR group was significantly decreased compared with that of the SA and PRE groups (*p* < 0.001). Compared with the TOR group, the expression level of hepcidin in the liver of the IBA and POST groups was markedly increased (+58.4% and +27.1%; *p* < 0.001, *p* = 0.048, respectively). These results suggest that hepcidin expression first decreased then increased during hibernation, except for the IBA group. The expression level of hepcidin in the POST group was lower than that in the SA groups (*p* = 0.038) (Figure 7A). Results also showed that HJV expression in the liver was significantly lower in the TOR group than in the SA group (−34.3%, *p* = 0.018). In addition, HJV expression in the IBA group was significantly higher than that in the TOR group and substantially higher than that in the NP group (*p =* 0.018, *p* = 0.036, respectively) (Figure 7B). Results also showed that compared with the other groups, BMP-6 expression was lowest in the TOR group and significantly increased in the IBA group. Compared with the TOR group, expression levels in the NP and POST group were substantially increased (+62.5%%, +91.6%; *p* = 0.029, *p* = 0.04, respectively, Figure 7C). In general, the expression levels of hepcidin, HJV, and BMP-6 in the liver showed similar trends.

### 2.5. Iron-Metabolism-Related Protein Expression in Femur during Different Periods

**Figure 8:**
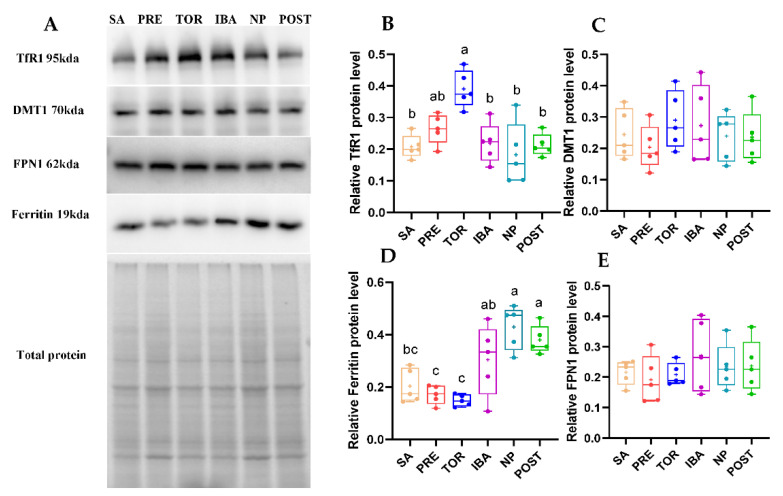
Changes in expression of iron-metabolism-related proteins in femur of Daurian ground squirrels during hibernation. (**A**) Western blot images of transferrin receptor 1 (TfR1), divalent metal transporter 1 (DMT1), ferritin, and ferroportin 1 (FPN1) in femur during hibernation (Figure S2, test 1). (**B**–**E**) Relative TfR1, DMT1, ferritin, and FPN1 protein expression in femur. SA, summer active group; PRE, pre-hibernation group; TOR, torpor group; IBA, interbout arousal group; NP, near post-hibernation group; and POST, post-hibernation group. Boxes represent upper and lower quartiles, middle horizontal line represents median, plus sign represents average, lines extending from upper and lower ends represent upper and lower edges, respectively, and points represent individual sample values (*n* = 5). Superscripts with different letters indicate significant differences (*p* < 0.05) among groups by Sidak test. Superscripts with same letters denote no significant differences (*p* > 0.05).

The expression levels of TfR1, DMT1, ferritin, and FPN1 in the squirrel femurs during hibernation can reflect the status of iron absorption, storage, and release. Here, the expression levels of DMT1 and FPN1 showed no significant differences among groups (Figure 8C,E). The expression of TfR1 in the TOR group was significantly higher than that in the SA, IBA, NP, and POST groups (+90.1%, +80.9%, +105.4%, and 76.7%; *p* = 0.001, *p* = 0.002, *p* < 0.001, and *p* = 0.001, respectively) (Figure 8B). These findings suggest that iron absorption capacity in the femur was enhanced in the TOR period. Compared with the SA, ferritin expression in the TOR group showed a downward trend (*p* = 0.072). Compared with the TOR group, ferritin expression in the IBA, NP, and POST groups was markedly increased (+126.2%, +173.3%, and +153.1%; *p* = 0.034, *p* = 0.019, and *p* = 0.005, respectively) (Figure 8D), indicating improvement in iron storage capacity.

## 3. Discussion

Iron overload is an independent risk factor for disuse osteoporosis [7,16,25]. However, whether prolonged inactivity in hibernating animals induces disturbance in iron metabolism and bone structure and function is unclear. In the current study, from the perspective of iron metabolism, we explored the resistance of hibernating Daurian ground squirrels to disuse osteoporosis by measuring the microstructure and mechanical strength of the femur in different periods, as well as changes in iron content and homeostasis-related proteins.

Based on microstructure and mechanical strength analysis of the femur, results showed no significant differences in ultimate stress and stiffness in the different hibernation periods, suggesting that the bone mechanical properties of Daurian ground squirrels are effectively maintained during long-term inactivity. This is consistent with earlier reports as well as previous studies on yellow-bellied marmots and thirteen-lined ground squirrels [21,26,27]. Our results also showed no remarkable changes in the femur mass of Daurian ground squirrels during hibernation, although femoral Tb.Sp was significantly higher in the POST group than in the SA group, suggesting bone loss. Notably, there were no significant differences in TMD, Ct.Ar, or CT.Ar/Tt.Ar in the POST group. These results are consistent with previous research on woodchucks (*Marmota monax*), which show no reduction in cortical BMD, apparent area, or bone area fraction, but slight bone trabecular loss during hibernation [28]. Thus, these findings indicate that hibernating animals maintain good bone mechanical strength and cortical bone microstructure during hibernation, with only slight cancellous bone loss.

The liver is an important organ for iron storage in the body, and iron concentrations in the liver are considered the gold standard for evaluating iron content in the body [29]. In this study, compared with the SA group, iron content in the femur decreased significantly in the TOR group (*p* < 0.05), consistent with our previous pilot study [30]. This low iron state of squirrels in the TOR group, which may be a survival adaptation to avoid iron-overload-induced bone loss caused by long-term disuse and to maintain motor function to cope with the harsh environment. According to a previous study, serum iron content in brown bears (*Ursus arctos*) during hibernation is significantly lower than that in spring and summer [31], suggesting that low iron content in tissues during hibernation may be a common strategy.

Notably, iron content in the liver and femur of the NP group was restored to the level of the SA group during the same inactive and fasting state of hibernation. Thus, iron metabolism showed extraordinary plasticity, suggesting that iron redistribution may occur in Daurian ground squirrels. Previous research has shown that liver iron content in male brown bears is three times higher during hibernation [32], suggesting that altered distribution of iron content in tissues during hibernation may be a common strategy to cope with extreme environments. In addition, the Prussian blue staining results indicated that iron distribution in the liver and femur was consistent with the changing trends in iron content. Although the liver and femur were in a low iron state in the TOR group, iron content increased in both in the IBA group, similar to that in the SA group. Iron content showed periodic fluctuation in the TOR and IBA groups, again demonstrating iron metabolism plasticity.

The reversible low iron state observed in Daurian ground squirrels during prolonged inactivity is radically different from the tissue iron overload found in non-hibernating animals in the state of disuse. For example, iron deposition occurs in the femur and liver of mice after hind limb unloading (28 days) [17]. Humans subjected to bed rest for 5 days under dry immersion (simulated microgravity) show an increase in spleen iron concentration and serum iron and transferrin saturation [33]. As iron overload is an independent risk factor for osteoporosis, we hypothesized that iron homeostasis plasticity is an important mechanism to avoid iron overload and prevent disuse osteoporosis in Daurian ground squirrels during hibernation.

Hepcidin is an important regulatory factor of systemic iron homeostasis secreted by the liver, which controls systemic iron content by inhibiting FPN1 expression in small intestinal epithelial cells, hepatocytes, and reticuloendothelial macrophages [34] (Figure 1A). Under tissue iron overload, hepcidin expression increases, which reduces the entry of intracellular iron into systemic iron; correspondingly, hepcidin expression decreases under iron deficiency [35]. In this study, hepcidin expression first decreased and then increased during hibernation, consistent with the change trend in iron content. These results suggest that the liver and femur were in a low iron state in the TOR group, and the corresponding expression level of hepcidin was down-regulated. Down-regulation of hepcidin can reduce the inhibition of iron entering the blood system to increase system iron reserves and avoid effects on tissues under low levels. However, the expression of hepcidin was significantly up-regulated with recovery of iron content post-hibernation. At the same time, hepcidin expression in the IBA group was consistent with the iron content trend in the same period, comparable to that in the SA group and significantly higher than that in the TOR group. The changes in hepcidin expression further confirmed the low iron state of the body during hibernation, as well as the plasticity and cyclical changes in iron metabolism.

In addition to the feedback regulation of iron content, hepcidin expression is also affected by the joint action of BMPs and hepcidin regulator HJV (a co-receptor and signal of BMPs) [36,37], with the SMAD signaling pathway activated to promote hepcidin transcription when BMPs bind to HJV [38,39]. In the current study, we found that changes in HJV and BMP-6 expression in the liver during different periods of hibernation were very similar to that of hepcidin.

The low iron content and hepcidin expression observed in Daurian ground squirrels during prolonged disuse inactivity differ significantly from the iron overload and hepcidin up-regulation found in non-hibernating animals under simulated weightlessness and disuse. For example, hepcidin content in the liver and iron deposition in the liver and bone are significantly increased in mice after hindlimb unloading for 28 days [13]. Hepcidin mRNA expression in the liver is significantly up-regulated in rats after hindlimb unloading for 7 or 14 days [40,41]. Thus, changes in the expression levels of hepcidin and its upstream proteins (BMP6 and HJV) appear to be another important mechanism for iron metabolism plasticity in hibernating Daurian ground squirrels.

Maintenance of intracellular iron homeostasis is affected by the expression of iron absorption proteins TfR1 and DMT1, iron storage protein ferritin, and iron-releasing protein FPN1 [42] (Figure 1B). DMT1 is responsible for iron release from endosomes to the cytoplasm. As the only known iron-releasing protein in vertebrates, FPN1 exports intracellular free iron to enter circulation and plays an important role in regulating iron homeostasis [43]. Here, western blot analysis showed that no significant differences in the expression levels of DMT1 and FPN1 among the study groups. This suggests that DMT1 and FPN1 may not be involved in plasticity changes in bone iron metabolism in Daurian ground squirrels during hibernation.

TfR1 mediates cellular iron uptake and endocytosis. When tissue iron reserves are deficient, the expression of TfR1 is increased to improve iron uptake, thereby balancing cellular iron homeostasis [44]. Compared with the SA and POST groups, femoral TfR1 expression increased significantly in the TOR group (*p* < 0.05), suggesting that the intracellular iron reserve was low, thus confirming the low iron content during this period. This is consistent with a previous proteomic study on yellow-bellied marmots, showing that the expression of TfR1 is noticeably up-regulated in bones during the hibernation period compared with the summer active period [23]. These results suggest that iron absorption capacity in the bone tissue of hibernating animals may increase during hibernation. It has been reported that the markedly up-regulated expression levels of TfR1 and DMT1 are the main mechanisms leading to iron overload in the femurs of hindlimb-suspended mice (28 days) [13]. In contrast, TfR1 protein expression in the TOR group was significantly up-regulated in Daurian ground squirrels, while femoral iron content was significantly decreased. On the basis of the above report and our results, we speculate that up-regulation of TfR1 protein expression in the femur may be caused by the negative feedback up-regulation of low iron content in the TOR group. This regulation also allows advanced preparation for the bones to reserve iron to ensure motor function post-hibernation.

Ferritin is an important iron storage protein in the body, and its cage-like structure is beneficial for inducing iron oxidation and deposition, thereby protecting cells from oxidative damage caused by excess iron [45,46]. When intracellular iron levels are low, iron regulatory proteins (IRPs) regulate mRNA-encoding ferritin at the transcriptional stage to decrease ferritin expression, and vice versa [47]. The initial decrease and subsequent increase in iron content is a direct factor resulting in corresponding changes in ferritin expression level. Expression of ferritin in the femur decreased in the TOR group but increased in the IBA and NP groups, thus reflecting the plasticity of tissue iron storage capacity.

## 4. Materials and Methods

### 4.1. Animals and Groups

Daurian ground squirrels were captured in Weinan City, Shaanxi Province, China. These small mammals are known to hibernate each year but appear to resist disuse-induced osteoporosis [48]. All animal-related experiments were approved by the Animal Experiment Ethics Committee of Northwest University (License No: SYXK 2010-004). Adult squirrels are captured from April to May each year and maintained in an animal feeding room (temperature set to 18–25 °C) with free access to food and water. After 3–4 weeks of environmental adaptation, according to different seasons and hibernation states, the ground squirrels were divided into the following six groups (male-to-female ratio (F:M) of approximately 2:1): summer active group (SA), pre-hibernation group (PRE), torpor group (TOR), interbout arousal group (IBA), near post-hibernation group (NP), and post-hibernation group (POST). Squirrels in the SA and PRE groups were raised under a natural light–dark cycle until sampling. The other groups entered the hibernation state in mid- to late-November of that year, at which time they were transferred to a hibernation room (dark state) at a temperature of 4–8 °C. During hibernation, body temperature (Tb) was monitored using an infrared thermal imager at 9:00 am and 9:00 pm every day, and animal state was observed under weak light. Details on the state of animals in different groups are shown in Table 1.

### 4.2. Sample Collection and Preparation

The Daurian ground squirrels were anesthetized with pentobarbital sodium (90 mg/kg) and placed on a thermostat control autopsy table. Femur and liver samples were collected, weighed, and recorded. The samples were quickly frozen with liquid nitrogen and stored in a refrigerator at −80 °C for later use. The animals were euthanized with an anesthetic overdose after operation.

### 4.3. Micro-Computed Tomography (CT)

The femurs were scanned ex vivo using a micro-CT scanner (Quantum GX2, PerkinElmer, Waltham, MA, USA) with an X-ray tube potential of 50 kVp and tube current of 80 μA in air. The captured images were acquired at a voxel size of 43 μm (filter: Cu 0.06 mm + Al 0.5 mm). Region of interest (ROI) was defined as the coronal central region of the distal femur (0.5 mm thick; number of slices: 512 layers). Selection of ROIs was determined using threshold segmentation, which distinguished cortical and cancellous bone. Relevant parameters of cortical and cancellous bone were collected [26]. Cortical parameters included cortical area (Ct.Ar, mm2), cortical area fraction (Ct.Ar/Tt.Ar, %), and average cortical thickness (Ct.Th, mm). Trabecular parameters included tissue mineral density (TMD, mg/cm^3^), bone volume fraction (BV/TV, %), trabecular number (Tb.N, 1/mm), trabecular separation (Tb.Sp, mm), and trabecular thickness (Tb.Th, mm).

### 4.4. Mechanical Properties

Right femurs were removed from the refrigerator (–80 °C), thawed under ambient temperature, and placed on a three-point bending mechanical tester (PLD-5, SANS, China) (Figure 3A), under the parameters: speed 0.5 mm/min, span 16 mm, and support radius 5 mm. The test was completed when the sample broke (ultimate force, F), and the outer axis, outer minor axis, inner major axis, and inner minor axis of the hollow ellipse (a_0_, b_0_, a_i_, and b_i_, respectively) of the fractured section were measured with a calibrated caliper. The measured indices included stiffness (S, N/mm) and ultimate stress (δ, MPa), with S calculated as the slope of the linear proportion of the load–deformation curve [24] and δ calculated using Formulas (1) and (2).

δ = FLb_0_/4I (1)

I = *π* (a_o_b_o_^3^ − a_i_b_i_^3^)/4 (2)

### 4.5. Iron Measurements

Femur and liver samples were thawed naturally, then placed in a crucible and dried in an oven at 80 °C for 48 h. Dried samples (0.1 g) were placed in a muffle furnace (420 ± 2 °C). After complete carbonization, the samples were naturally cooled and then dissolved in aqua regia to obtain a clear solution. Iron ion standard curves were prepared, and total iron content in the samples was detected using an inductively coupled plasma spectrometer (iCAP 6300, Thermo Scientific, Waltham, MA, USA).

### 4.6. Histological Analysis

Liver samples were cut into 5 × 5-mm tissue blocks and fixed with 4% formaldehyde solution for 24 h. Femoral tissue was fixed with 4% formaldehyde for 48 h, then treated with EDTA decalcification solution until the bone tissue softened. The liver and femur tissue samples were processed, including dehydration, paraffin embedding, and sectioning (RM2016, Shanghai Leica Instrument Co., Ltd., Shanghai, China). The sections were stained with Prussian blue and incubated with diaminobenzidine (DAB) for 25 min. After scanning, the sections were analyzed using Image-Pro Plus v6.0.

### 4.7. Enzyme-Linked Immunosorbent Assay (ELISA)

Each femur was weighed, cut into pieces, and placed in a centrifugal tube. A corresponding volume of tissue lysis fluid was added to each tube (1:9 weight/volume ratio), and the sample was placed in an ice bath and completely cracked with a homogenizer. The resulting homogenate was centrifuged at 5000 rpm for 10 min at 4 °C, and the extracted protein supernatant was collected and stored in a refrigerator at −80 °C. Hepcidin, hemojuvelin (HJV), and bone morphogenetic protein 6 (BMP-6) were measured using an ELISA kit (Shanghai Jianglai Biotech, Shanghai, China), following the manufacturer’s instructions.

### 4.8. Western Blot Analysis

In a liquid nitrogen environment, femoral samples were ground into powder, and 10 times the volume of RIPA lysis buffer was added for tissue homogenization in an ice bath. After that, the supernatant was obtained by centrifugation at 15,000× *g* rpm for 15 min at 4 °C. The protein content in the supernatant was detected using a protein detection kit (Thermo Fisher Scientific, 23227, USA). The supernatant was mixed with a loading buffer at a ratio of 1:4 *v*/*v* to adjust the protein concentration to 2.5 μg/μL. The prepared samples were boiled for 10 min and stored at −80 °C until use.

At 120 V constant voltage, 10% sodium dodecyl sulfate-polyacrylamide gel electrophoresis (SDS-PAGE) was used for protein separation. The proteins were then transferred to 0.45-μm aperture polyvinylidene fluoride membranes using the wet transfer method [49]. The membranes were then incubated with 5% bovine serum albumin solution for 2 h at room temperature, and then with primary antibodies overnight at 4 °C, including transferrin receptor 1 (TfR1) (1:1000) (Santa, Santa Cruz, USA), ferritin (1:1000) (Proteintech, Wuhan China), divalent metal transporter 1 (DMT1) (1:1000) (Proteintech, China), and ferroportin 1 (FPN1) (1:1000) (Abcam, Cambridge, UK). The following day, the membranes were washed three times with TBST (Tris HCl, 150 mM NaCl, 0.05% Tween-20, pH 7.5, 10 min each time), then incubated with secondary antibodies (horseradish peroxidase (HRP)-conjugated anti-rabbit or anti-mouse antibodies, Zhuangzhi, Xi’an, China) at room temperature for 2 h, followed by chemiluminescence detection (Thermo Fisher Scientific, NCI5079, Eugene, OR, USA) and washing with TBST. According to the manufacturer’s instructions, the gray value of each lane after removing background gray value was detected by Image-Pro Plus 6.0. Total protein staining of the gel was used as a normalized control for all blots, as per pervious research [49]. Relative expression of the target protein was expressed as a ratio of the gray value of the sample to the gray value of the total protein in the corresponding lane in the gel.

### 4.9. Statistical Analysis

SPSS v17.0 was used for all statistical tests. One-way analysis of variance (ANOVA) was used for comparisons between two groups. Tamhane (Levene, *p* < 0.05) and Sidak (Levene, *p* > 0.05) tests were used for multiple range tests of the data (Table S2). Here, *p* < 0.05 was considered statistically significant. Data in the figures are expressed as individual sample values, upper and lower quartiles, and average, median, and upper and lower edges are reported.

## 5. Conclusions

**Figure 9:**
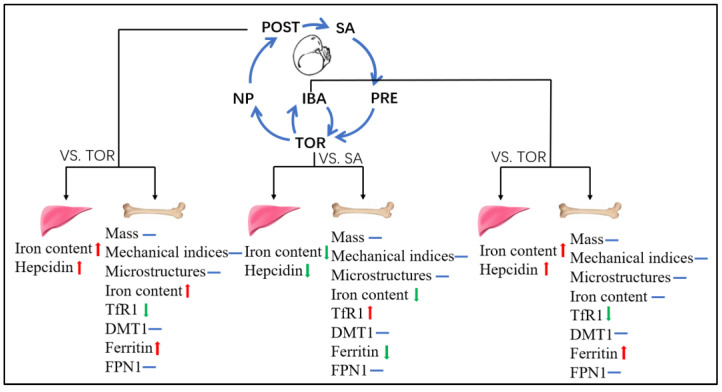
Plasticity changes in iron-metabolism-related indices during hibernation. (1) In TOR group (TOR vs. SA), iron content in liver and femur decreased, hepcidin expression decreased, iron storage capacity in the femur decreased, and iron absorption capacity increased; (2) In IBA group (IBA vs. TOR), iron content and hepcidin expression in the liver increased, and iron storage capacity in the femur increased; (3) In POST group (POST vs. TOR), iron content in the liver and femur increased (recovery), and hepcidin expression increased; iron storage capacity in the femur increased, and iron absorption capacity decreased. SA, summer active group; PRE, pre-hibernation group; TOR, torpor group; IBA, interbout arousal group; NP, near post-hibernation group; and POST, post-hibernation group. Note: red arrow indicates up-regulation, green arrow indicates down-regulation, and blue line indicates no significant difference.

The femur of Daurian ground squirrels was well-maintained after long-term inactivity (148 ± 6 days) and exhibited only limited cancellous bone loss. Changes in iron and ferritin content in the femur initially decreased and then increased during hibernation, indicating extraordinary bone iron metabolism plasticity. The periodic return of liver iron content and its negative regulator hepcidin to normal levels during interbout arousal may be a protective strategy to avoid low iron reserves during hibernation and again reflects considerable iron metabolism plasticity (Figure 9). Thus, the plasticity of bone iron metabolism during hibernation is the most important discovery of this study. The reversible low iron state during hibernation may be a major mechanism to avoid disuse-induced iron overload and bone loss. In addition, the results also suggest that manipulating the storage or redistribution of iron in the body may be a new idea to prevent disuse-induced iron overload; however, since the mechanisms behind this remarkable ability of hibernating Daurian ground squirrels have not been studied, the implementation of translatable application to humans and the clinical application of this new idea need further work.

## 6. Limitations

In this study, Daurian ground squirrels exhibited extraordinary plasticity in iron metabolism during hibernation. However, due to the depletion of annual samples, we did not explore the redistribution of iron content in this study. Therefore, on the basis of existing results and improvement in experimental design, we will further study the tissues (e.g., spleen, blood, cardiac muscles, and skeletal muscle) involved in iron redistribution and the underlying mechanism. In addition, this study focused on Daurian ground squirrels from a single area (Weinan City, Shaanxi Province), and whether all squirrels exhibit bone iron metabolism plasticity needs to be studied across a wider region.

## Figures and Tables

**Table 1:** Animal groups and sample times. Animals were divided into six groups and sampled between June of that year and March of the following year.

Group	Sample Time	Tb of Animal
SA	Mid-June	>37 °C
PRE	Mid-September	>37 °C
TOR	After two months hibernation, animals enter a new hibernation bout	≥5 days with stable Tb of 5–8 °C
IBA	Same as TOR	34–37 °C for less than 12 h
NP	March of following year	5–8 °C for less than 24 h
POST	Same as NP	36–38 °C for more than 3 d

Note: SA, summer active group; PRE, pre-hibernation group; TOR, torpor group; IBA, interbout arousal group; NP, near post-hibernation group; and POST, post-hibernation group.

## Data Availability

The data that support the findings of this study are available from the corresponding author upon reasonable request.

## Supplementary Materials

The following supporting information can be downloaded at: https://www.mdpi.com/article/10.3390/ijms232415858/s1.
