# Supplementary material for: Remarkable Plasticity of Bone Iron Homeostasis in Hibernating Daurian Ground Squirrels (Spermophilus dauricus) May Be Involved in Bone Maintenance
Source: Int J Mol Sci. 2022 Dec 13;23(24):15858. doi: 10.3390/ijms232415858 (PMC9779590; doi:10.3390/ijms232415858)
Supplement: Supplementary file 1 [file ijms-23-15858-s001.zip › ijms-1918080-supplementary.pdf]

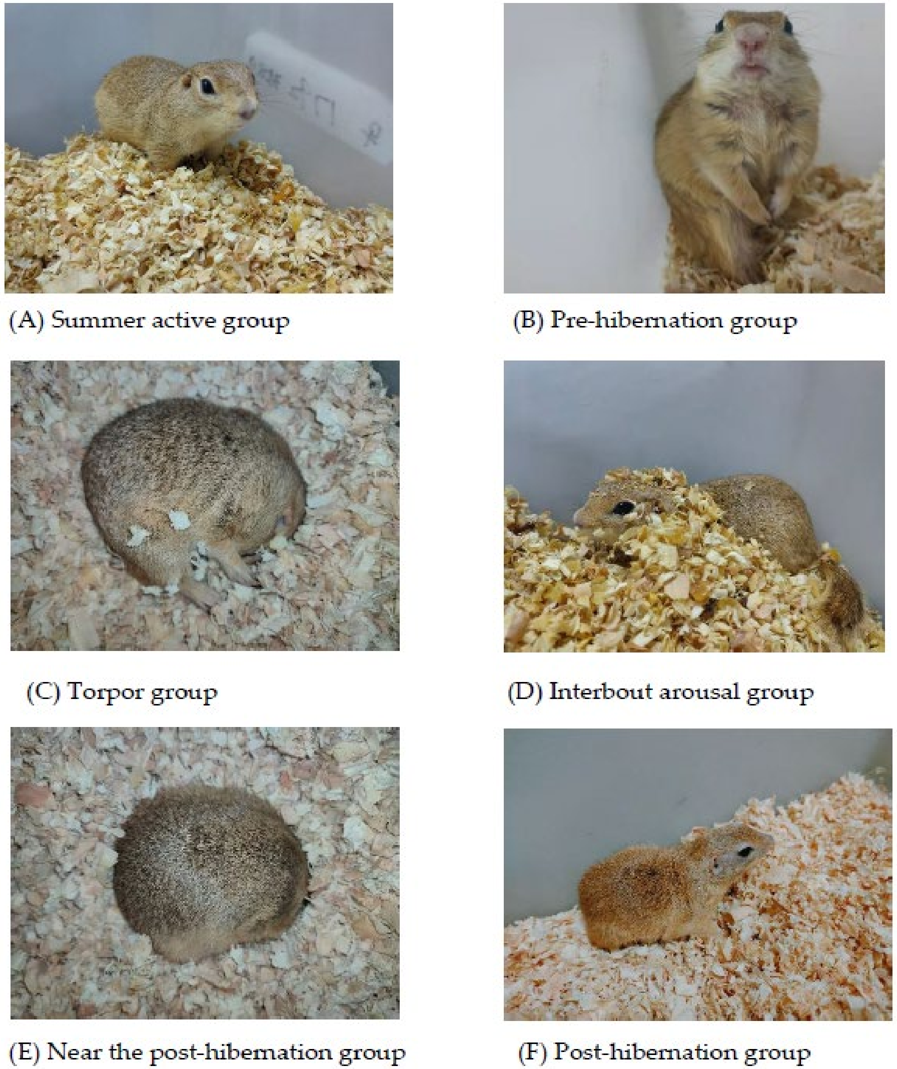

Figure S1. State of Daurian ground squirrels at different stages of hibernation. (A) Summer active group, (B) pre-hibernation group, (C) torpor group, (D) interbout arousal group, (E) near the post-hibernation group, (F) post-hibernation group. The entire hibernation period consists of multiple Torpor (at least 5 d with a stable body temperature of 5–8 °C) and Interbout arousal (34-37 °C for less than 12 h) cycles.

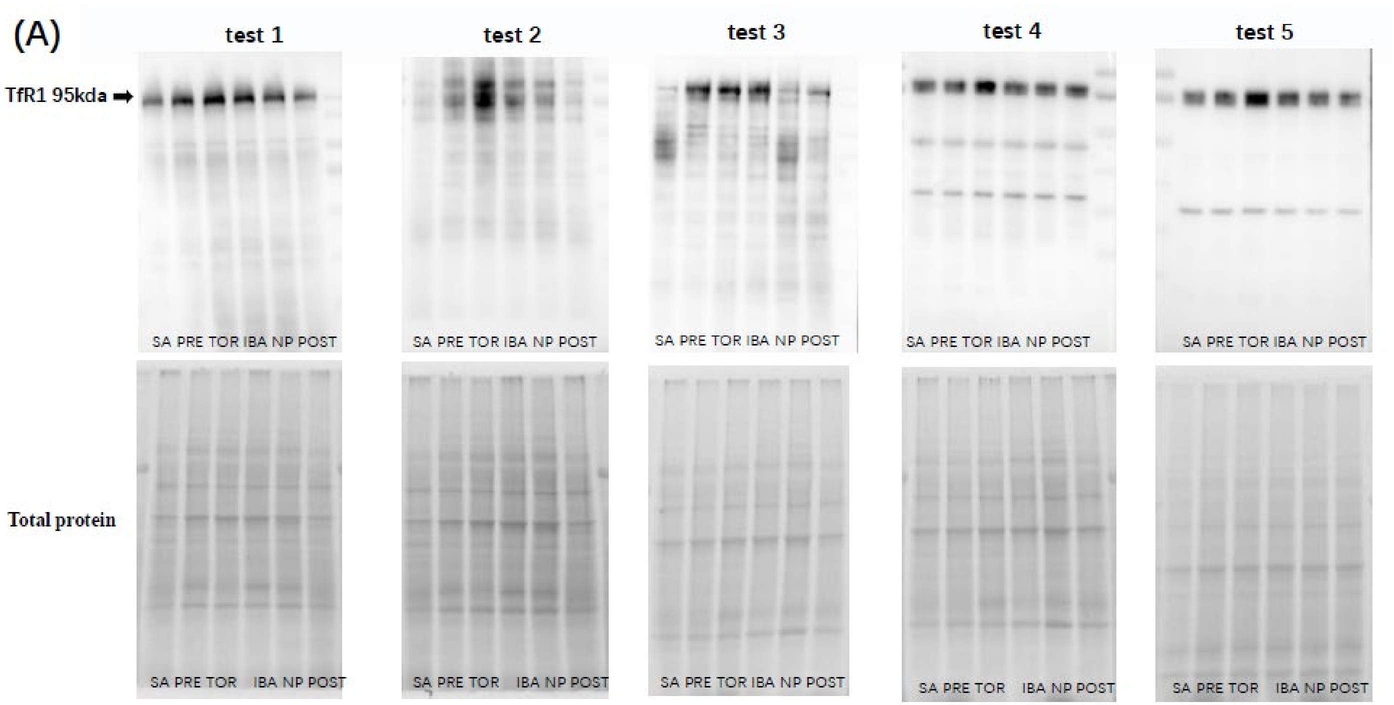

(A)
test 1
test 2
test 3
test 4
test 5
TfR1 95kda
SA PRE TOR IBA NP POST
Total protein
SA PRE TOR IBA NP POST

(B)

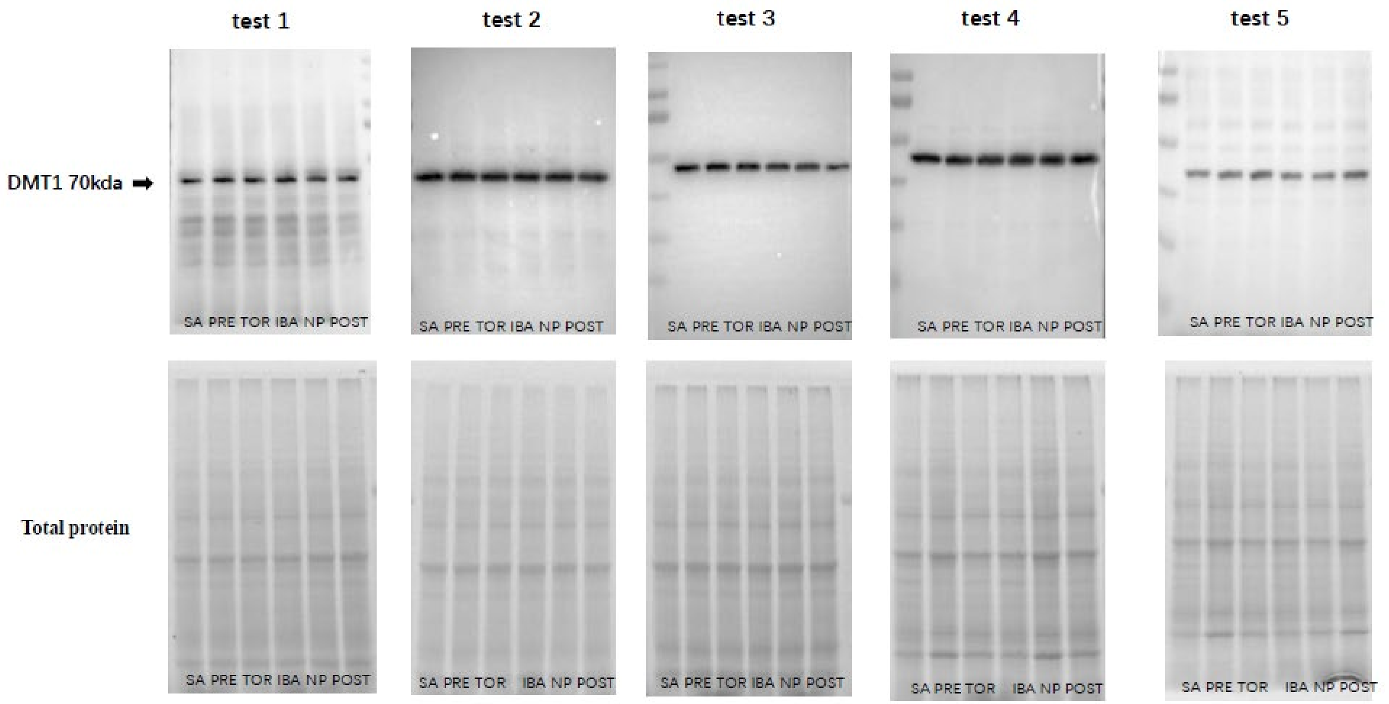
test 1
test 2
test 3
test 4
test 5
DMT1 70kda
Total protein
SA PRE TOR IBA NP POST

(C)

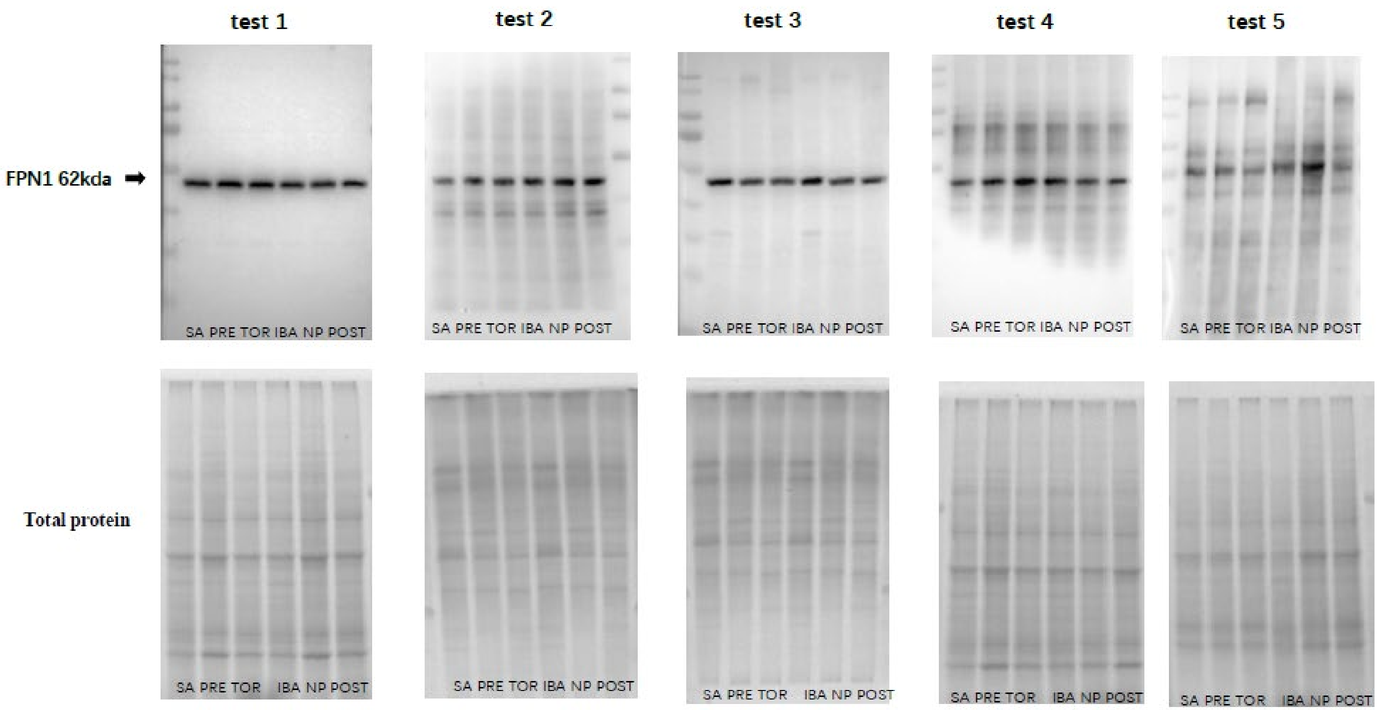
test 1
test 2
test 3
test 4
test 5
FPN1 62kda
SA PRE TOR IBA NP POST
SA PRE TOR IBA NP POST
SA PRE TOR IBA NP POST
SA PRE TOR IBA NP POST
SA PRE TOR IBA NP POST
Total protein
SA PRE TOR IBA NP POST
SA PRE TOR IBA NP POST
SA PRE TOR IBA NP POST
SA PRE TOR IBA NP POST
SA PRE TOR IBA NP POST

(D)

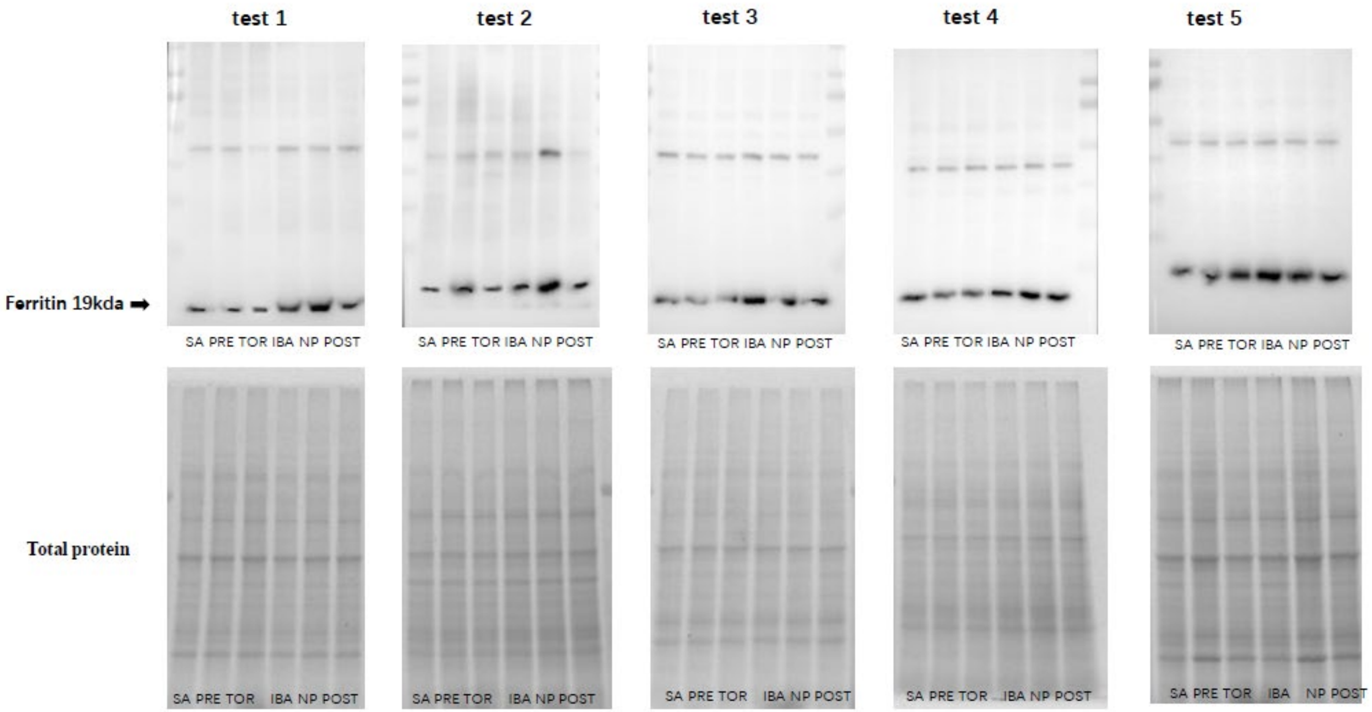

Figure S2. Western blotting of iron metabolism-related proteins in femur of Daurian ground squirrels during hibernation (n = 5). (A) TfR1, (B) DMT1, (C) FPN1, (D) Ferritin. Notes: TfR1, Transferrin receptor 1; DMT1, divalent metal transporter 1; FPN1, Ferroportin 1. SA, summer active group; PRE, pre-hibernation group; TOR, torpor group; IBA, interbout arousal group; NP, near post-hibernation group; and POST, post-hibernation group.

Table S1. The results of Micro CT in femur of Daurian ground squirrels in different periods. Cortical parameters included cortical area (Ct.Ar, mm2), cortical area fraction (Ct.Ar/Tt.Ar, %), and average cortical thickness (Ct.Th, mm). Trabecular parameters included tissue mineral density (TMD, mg/cm3), bone volume fraction (BV/TV, %), trabecular number (Tb.N, 1/mm), trabecular separation (Tb.Sp, mm), and trabecular thickness (Tb.Th, mm). Notes: SA, summer active group; PRE, pre-hibernation group; TOR, torpor group; IBA, interbout arousal group; NP, near post-hibernation group; and POST, post-hibernation group.

	**SA**	**PRE**	**TOR**	**IBA**	**NP**	**POST**
TMD (mg/cm3)	3134.68	3012.05	2919.26	2795.88	3076.95	2792.45
	2791.29	2515.98	2814.17	2992.49	2558.99	2968.81
	3002.23	2900.8	3201.23	2801.34	2724.78	3100.11
	2711.36	3101.99	2610.31	2908.41	2790.82	2543.21
	2509.4	2693.44	2708.66	3001.55	2529.78	3015.93
	2694.1	3005.24	2899.51	2460.33	2807.59	2400.88
	**SA**	**PRE**	**TOR**	**IBA**	**NP**	**POST**
BV/TV (%)	32. 95	24. 2	24. 54	32. 13	29. 97	33. 37
	27. 4	32. 11	28. 44	33. 18	25. 43	32. 46
	31. 4	28. 4	29. 22	30. 4	26. 83	30. 1
	25. 6	27. 3	31. 55	25. 2	23. 98	25. 7
	26. 4	35. 24	25. 6	25. 1	23. 16	26. 7
	26. 2	27. 6	28. 5	24. 6	31. 27	29. 9
	**SA**	**PRE**	**TOR**	**IBA**	**NP**	**POST**
Tb.Th (mm)	0. 0795	0. 0789	0. 0642	0. 0718	0. 0775	0. 0775
	0. 0729	0. 0668	0. 0707	0. 0609	0. 082	0. 082
	0. 0829	0. 0728	0. 0722	0. 0577	0. 072	0. 072
	0. 071	0. 0618	0. 0787	0. 0609	0. 0662	0. 0662
	0. 0649	0. 0632	0. 0607	0. 0667	0. 0702	0. 0702
	0. 0769	0. 0774	0. 0647	0. 0784	0. 0632	0. 0632
	**SA**	**PRE**	**TOR**	**IBA**	**NP**	**POST**
Tb.Sp (mm)	0. 1449	0. 1914	0. 1728	0. 1908	0. 1562	0. 2251
	0. 1319	0. 1814	0. 1815	0. 1719	0. 1755	0. 1923
	0. 1377	0. 1828	0. 1722	0. 1821	0. 1863	0. 2013
	0. 1619	0. 1788	0. 1671	0. 1687	0. 1901	0. 1613
	0. 1818	0. 1523	0. 1815	0. 1887	0. 1431	0. 1713
	0. 1421	0. 1691	0. 1515	0. 1776	0. 1976	0. 1813
	**SA**	**PRE**	**TOR**	**IBA**	**NP**	**POST**
Tb.N (1/mm)	1. 6669	1. 9191	1. 5242	1. 7563	1. 9309	2. 1790
	1. 4591	2. 0502	1. 9174	2. 0374	1. 0723	1. 5933
	1. 5541	2. 1102	1. 8174	1. 8984	1. 7605	1. 7933
	1. 6591	1. 9702	1. 8554	2. 0374	1. 6971	1. 8633

	1. 4123	2. 0002	1. 7774	1. 8274	1. 9975	1. 7733
	1. 4545	1. 8302	1. 93	1. 5974	1. 6955	1. 6833
	**SA**	**PRE**	**TOR**	**IBA**	**NP**	**POST**
Ct.Th (mm)	0. 1881	0. 1888	0. 1951	0. 1904	0. 1986	0. 2037
	0. 1792	0. 1931	0. 1925	0. 1917	0. 1906	0. 1906
	0. 1912	0. 1882	0. 1945	0. 1806	0. 1955	0. 1955
	0. 1732	0. 1761	0. 1925	0. 1748	0. 1936	0. 1936
	0. 1752	0. 1821	0. 1785	0. 1878	0. 2037	0. 1986
	0. 1988	0. 1771	0. 1811	0. 1952	0. 1816	0. 1916
	**SA**	**PRE**	**TOR**	**IBA**	**NP**	**POST**
Ct.Ar/Tt.Ar (%)	16. 7608	19. 7623	20. 8258	19. 6814	22. 6895	20. 039
	16. 6355	18. 2434	16. 6194	19. 1462	22. 5271	18. 6759
	15. 5102	17. 7245	18. 6124	18. 611	21. 9423	17. 3128
	17. 3849	16. 5056	19. 6193	18. 0758	16. 885	18. 9497
	15. 2596	18. 6867	16. 2144	17. 2806	19. 0101	17. 5866
	13. 4343	19. 1678	18. 2564	17. 9154	21. 5154	18. 1235
	**SA**	**PRE**	**TOR**	**IBA**	**NP**	**POST**
Ct.Ar （mm2)	2. 3233	1. 9854	2. 3129	2. 3996	2. 3322	2. 3322
	2. 2717	1. 9458	2. 2703	2. 2091	2. 2085	2. 2085
	2. 3201	1. 9058	2. 4277	2. 2316	2. 3085	2. 3085
	2. 0985	2. 0658	2. 2351	2. 188	2. 1185	2. 1185
	2. 1369	2. 1958	2. 0125	2. 1029	2. 1785	2. 1785
	2. 4153	2. 1058	2. 1199	2. 4026	2. 2385	2. 2385

Table S2. *P* values of the differences between groups based on the multiple comparison tests (Tamhane or Sidak test). *P* values with statistical differences are marked in red. Notes: Ct.Ar (cortical area), Ct.Ar/Tt.Ar (cortical area fraction), Ct.Th (cortical thickness), TMD (tissue mineral density), BV/TV (bone volume fraction), Tb.N (trabecular number), Tb.Sp (trabecular separation), and Tb.Th (trabecular thickness). HJV, hemojuvelin; BMP6, bone morphogenetic protein 6; TfR1, Transferrin receptor 1; DMT1, divalent metal transporter 1; FPN1, Ferroportin 1. SA, summer active group; PRE, pre- hibernation group; TOR, torpor group; IBA, interbout arousal group; NP, near post-hibernation group; and POST, post-hibernation group.

Variables	Tissues	Groups														
		SA vs.					PRE vs.				TOR vs.			IBA vs.		NP vs.
		PRE	TOR	IBA	NP	POST	TOR	IBA	NP	POST	IBA	NP	POST	NP	POST	POST
Femur mass (g)	Femur	0.833	0.238	0.307	0.075	0.114	1.000	1.000	0.961	0.988	1.000	1.000	1.000	0.997	1.000	1.000
Ultimate stress (MPa)	Femur	1.000	1.000	0.867	1.000	0.993	1.000	0.965	1.000	0.953	0.985	0.999	0.911	0.530	0.168	1.000
Stiffness (N/mm)	Femur	0.888	1.000	1.000	1.000	0.993	0.410	0.994	0.821	0.183	0.989	1.000	1.000	1.000	0.876	0.998
TMD (mg/cm^3)	Femur	1.000	1.000	1.000	1.000	1.000	1.000	1.000	0.998	1.000	1.000	1.000	1.000	1.000	1.000	1.000
BV/TV (%)	Femur	1.000	1.000	1.000	1.000	1.000	1.000	1.000	0.979	1.000	1.000	1.000	0.999	0.999	1.000	0.891
Tb.Th (mm)	Femur	0.992	0.897	0.478	1.000	1.000	1.000	0.997	1.000	1.000	1.000	1.000	1.000	0.933	0.933	1.000
Tb.Sp (mm)	Femur	0.169	0.445	0.065	0.218	0.006	1.000	1.000	1.000	0.963	0.999	1.000	0.710	1.000	0.999	0.928
Tb.N (1/mm)	Femur	0.005	0.277	0.093	0.933	0.229	0.859	0.993	0.197	0.905	1.000	0.997	1.000	0.902	1.000	0.993
Ct.Th (mm)	Femur	1.000	0.993	1.000	0.405	0.193	0.992	1.000	0.397	0.188	1.000	0.991	0.898	0.822	0.541	1.000
Ct.Ar/Tt.Ar (%)	Femur	0.098	0.096	0.074	<0.001	0.075	1.000	1.000	0.130	1.000	1.000	0.134	1.000	0.170	1.000	0.168
Ct.Ar (mm^2)	Femur	0.021	1.000	1.000	1.000	1.000	0.073	0.027	0.070	0.070	1.000	1.000	1.000	1.000	1.000	1.000
Liver iron (μg/g)	Liver	0.001	0.017	1.000	1.000	1.000	0.988	0.001	<0.001	<0.001	0.014	0.008	0.012	1.000	1.000	1.000
Bone iron (μg/g)	Femur	0.449	<0.001	0.219	0.059	1.000	0.026	1.000	0.998	0.950	0.070	0.251	0.001	1.000	0.754	0.338
Prussian blue staining (IOD value)	Liver	0.149	0.010	0.947	0.813	1.000	0.856	0.451	0.800	0.157	<0.001	0.038	<0.001	1.000	0.836	0.757
Prussian blue staining of (IOD value)	Femur	<0.001	<0.001	0.001	<0.001	<0.001	0.676	0.089	0.515	0.931	0.001	0.008	0.042	0.999	0.878	1.000
Liver Hepcidin (ng/g)	Liver	1.000	<0.001	0.883	0.015	0.038	<0.001	0.997	0.058	0.140	<0.001	0.118	0.048	0.483	0.763	1.000
Liver HJV (ng/g)	Liver	0.119	0.018	1.000	0.038	0.945	0.709	0.011	0.917	0.460	0.018	1.000	0.076	0.036	0.538	0.164
Liver BMP6 (ng/g)	Liver	<0.001	<0.001	0.001	<0.001	<0.001	<0.001	0.540	0.889	0.816	<0.001	0.029	0.040	0.018	0.013	1.000
Relative protein level of DMT1 in bone	Femur	1.000	1.000	1.000	1.000	1.000	0.890	0.980	1.000	1.000	1.000	0.999	0.998	1.000	1.000	1.000
Relative protein level of TfR1 in bone	Femur	0.824	0.001	1.000	1.000	1.000	0.056	0.955	0.349	0.898	0.002	<0.001	0.001	0.998	1.000	1.000
Relative protein level of FPN1 in bone	Femur	1.000	1.000	0.991	1.000	1.000	1.000	0.838	0.999	0.999	0.971	1.000	1.000	1.000	1.000	1.000
Relative protein level of Ferritin in bone	Femur	0.953	0.072	0.141	0.035	0.009	0.928	0.046	0.016	0.003	0.034	0.019	0.005	0.387	0.711	0.995
